# The wide and growing range of lamin B-related diseases: from laminopathies to cancer

**DOI:** 10.1007/s00018-021-04084-2

**Published:** 2022-02-08

**Authors:** Camilla Evangelisti, Isabella Rusciano, Sara Mongiorgi, Giulia Ramazzotti, Giovanna Lattanzi, Lucia Manzoli, Lucio Cocco, Stefano Ratti

**Affiliations:** 1 Cellular Signalling Laboratory, Department of Biomedical and NeuroMotor Sciences (DIBINEM), University of Bologna, Bologna, Italy; 2 CNR Institute of Molecular Genetics “Luigi Luca Cavalli-Sforza”, Unit of Bologna, Bologna, Italy; 3 IRCCS Istituto Ortopedico Rizzoli, Bologna, Italy

**Keywords:** Lamin B, Nuclear lamina, Laminopathy, LMNB, Brain, Tumour

## Abstract

B-type lamins are fundamental components of the nuclear lamina, a complex structure that acts as a scaffold for organization and function of the nucleus. Lamin B1 and B2, the most represented isoforms, are encoded by *LMNB1* and *LMNB2* gene, respectively. All B-type lamins are synthesized as precursors and undergo sequential post-translational modifications to generate the mature protein. B-type lamins are involved in a wide range of nuclear functions, including DNA replication and repair, regulation of chromatin and nuclear stiffness. Moreover, lamins B1 and B2 regulate several cellular processes, such as tissue development, cell cycle, cellular proliferation, senescence, and DNA damage response. During embryogenesis, B-type lamins are essential for organogenesis, in particular for brain development. As expected from the numerous and pivotal functions of B-type lamins, mutations in their genes or fluctuations in their expression levels are critical for the onset of several diseases. Indeed, a growing range of human disorders have been linked to lamin B1 or B2, increasing the complexity of the group of diseases collectively known as laminopathies. This review highlights the recent findings on the biological role of B-type lamins under physiological or pathological conditions, with a particular emphasis on brain disorders and cancer.

**Graphical abstract:**

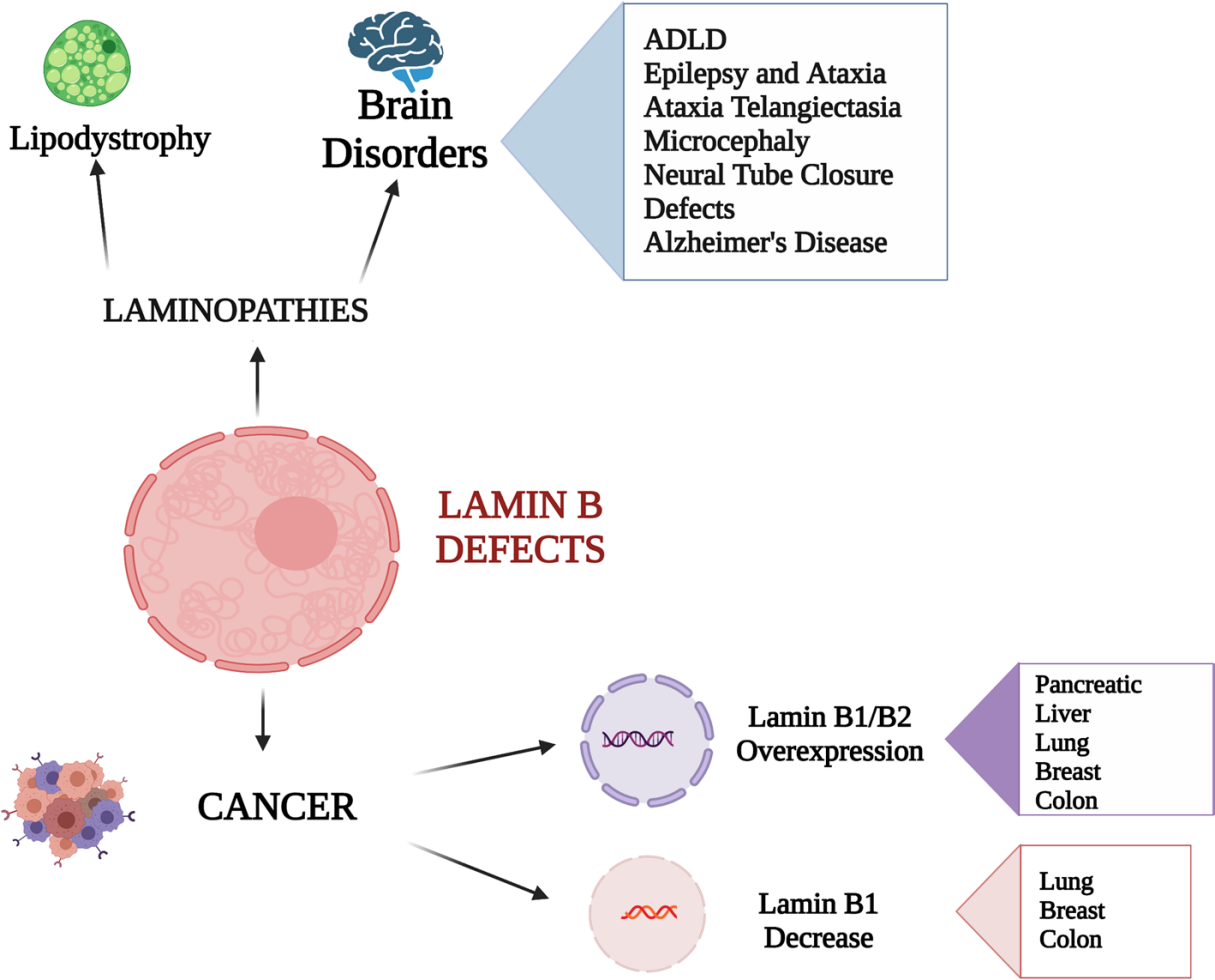

## Introduction

Lamins are type V intermediate filament proteins located beneath the inner nuclear membrane, where they form a high-ordered meshwork named nuclear lamina [1, 2]. The nuclear lamina provides structural support to the nucleus, contributing to nuclear shape and mechanical stability and supporting and regulating chromatin organization.

**Fig. 1:**
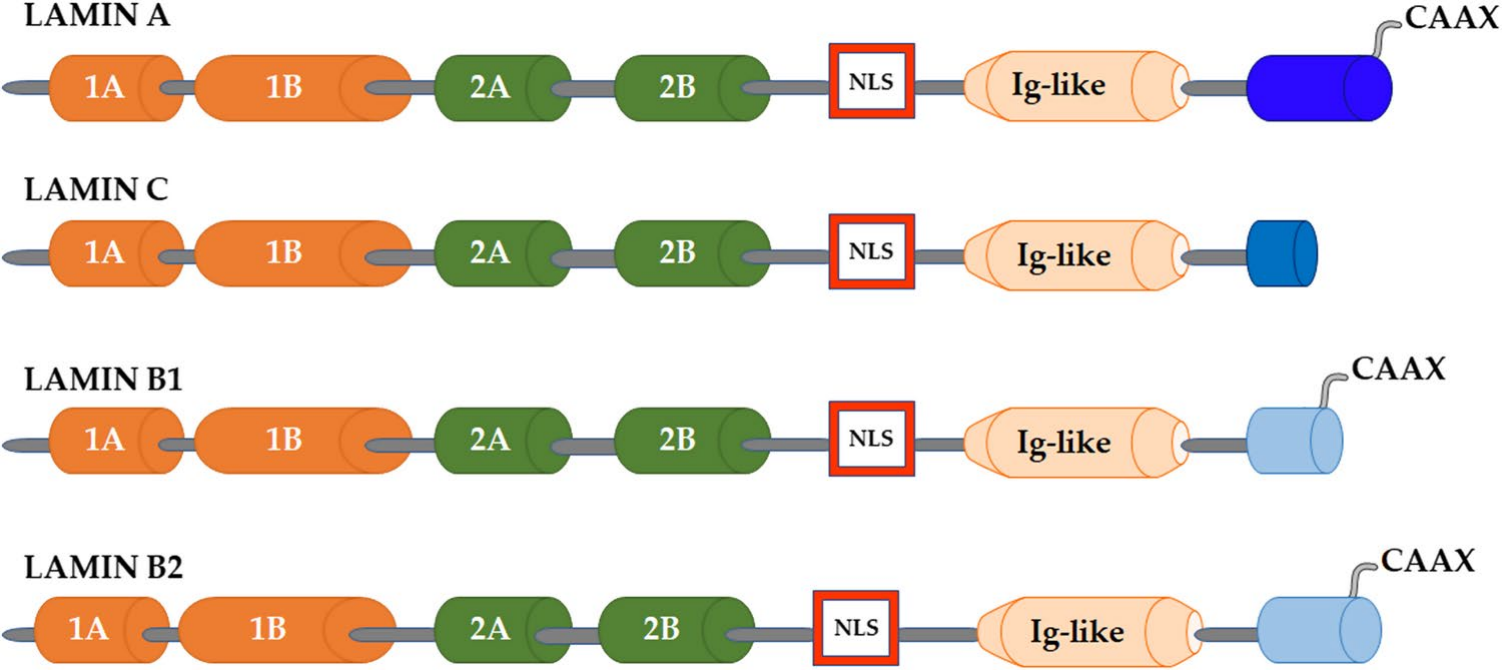
Domain organization of the main human lamin isoforms. The α-helical rod domain comprises four segments, 1A, 1B, 2A, 2B, which are separated by linker segments. The C-terminal tail domain contains a nuclear localization signal (NLS), an immunoglobulin domain (Ig-like), and a conserved CAAX box, which undergoes farnesylation

In mammals, lamins are divided into A- and B-types, based on their sequence homologies (Fig. 1).

*LMNA* gene, mapping on chromosome 1q11-q2, gives rise to different splicing isoforms: Lamin A, lamin C (the two major isoforms), lamin A ∆10 and lamin C2 [3]. Lamin A is translated as a precursor protein named prelamin A which is post-translationally modified. In particular, ZMPSTE24 metalloproteinase recognizes and cleaves the last 18 C-terminal aminoacids of farnesylated prelamin A, thus producing mature lamin A [3].

The two major B-type lamins, lamins B1 and B2, are encoded by different genes (*LMNB1* and *LMNB2*), located on chromosomes 5q23.2-q31.3 and 19p13.3, respectively (Fig. 1). *LMNB2* gene also encodes the minor isoform lamin B3, a germ cell-specific isoform that results from alternative splicing of the *LMNB2* gene [1]. All B-type lamins are translated as prelamins, requiring extensive post-translational modifications of the carboxy-terminal-CAAX box, which is subjected to farnesylation, carboxymethylation and partial cleavage to generate lamin B1 and lamin B2 mature forms [1]. The endoprotease involved in B-type lamin cleavage is the Ras-converting enzyme 1 (Rce1) [4]. While lamin B2 farnesylation is not essential for physiological development, this is not true for lamin B1 [5].

While A-type lamins are also present in the nucleoplasm as apparently freely diffusible molecules [6], B lamins are preferentially associated to the nuclear membrane, probably due to their permanently farnesylated state [7, 8]. Indeed, type-B lamins are also located in the nucleoplasm although they were less abundant and more static than type-A [7, 9, 10].

The different disassembly and assembly properties of A- and B-type lamins during mitosis suggest that lamins may be processed in different locations within the nuclear environment and that they form separate networks in the nuclear lamina [1, 8]. Indeed, the different lamin isoforms shape spatially separate but interacting overlapping filament meshwork [7, 9, 10]. Compared to lamin A and C, lamins B have a higher edge length and edge connectivity and in particular, lamin B1 has more edges per face than lamin B2 [9, 11].

The expression pattern is different between A- and B-type lamins. B lamins are expressed in most cell types, independently of their differentiation state, whereas lamin A/C is not expressed in embryonic stem cells, but in most differentiated cells [12].

B-type lamins are not mutually redundant during embryogenesis, being required for normal tissue development, in particular of the central nervous system (CNS) [13], and seem to be non-essential in some tissues, such as epidermis and liver [14]. Of note, lamin A (but not lamin C) is expressed at low levels in most neural and neuroendocrine cells, while lamin A/C is expressed upon stimulation in hematopoietic cells.

Regarding the protein structural properties, B-type lamin tetramers feature elastic properties, which allow significant deformation of the nuclear envelope [10, 11], while lamin A/C confers deformation-resistant viscous stiffness to nuclei [15]. Notably, a recent study by Wintner et al. showed that both lamin A/C and lamin B contribute to nuclear stiffness, while viscosity is specified mostly by lamin A [16].

Importantly, lamins A and B have different binding partners at the nuclear envelope and inside the nucleus, which is reflected in their involvement in a plethora of different intracellular pathways that affect both cytoskeleton and chromatin functional organization [7, 17, 18]. B1 and B2 have been shown to bind 23 and 7 protein partners, respectively [18], while they also bind chromatin at specific sequences called lamina-associated domains or LADs [19–21].

In this review, we will summarize and discuss the updated knowledge about the role of B-type lamins in physiological and pathological conditions. Considering the heterogenous and controversial field of lamin B-related diseases, the rarity of most of these pathologies, and non-completely understood mechanisms, this review is aimed at providing an overview of different disorders tethered by shared biological actors.

## Role of B-type lamins in cellular homeostasis

B-type lamins are involved in a wide range of nuclear functions, including structural support to the nucleus, regulation of chromatin and DNA replication, transcription and DNA repair [1]. In addition, lamins B are implicated in several cellular processes, including cell cycle regulation, cellular senescence, DNA damage, and tissue development.

### Cellular senescence

Lamin B1 is involved in cellular senescence, a cellular decline characterized by permanent cell cycle arrest and by a proinflammatory secretory phenotype (SASP) [22]. Lamin B1 loss causes alterations of nuclear shape and low lamin B1 levels are associated with geroconversion of cells, as it has been demonstrated in murine models and human tissues [23, 24].

Depletion of lamin B1 in senescent cells may be associated with changes in histone methylation [25, 26], including the reduction of repressive histone mark H3K27me3, and the activation of different SASP genes [26].

Freund and colleagues reported that, in human cells, reduced lamin B expression mediated by the p53 and pRb pathways is related to cellular senescence [24]. Authors observed that p53 and pRb activation is sufficient to reduce lamin B1 expression independently of known hallmarks of ageing, such as activation of DNA damage response (DDR), p38 mitogen-activated protein kinase (MAPK), nuclear factor-κB (NF-κB) and reactive oxygen species (ROS) [24]. On the other hand, in mice, lamin B1 deficiency is linked to an increase of ROS production through a dysregulation of different Oct-1-dependent genes that are involved in oxidative stress response [27].

A few years later, Dreesen et al. showed that the single lamin B1 depletion is not sufficient to trigger senescence; rather, it is likely that lamin B1 depletion modulates senescence when accompanied by additional stress, such as sparse growth [28]. The same authors observed that lamin B1 overexpression impairs proliferation and culminates in cellular senescence, with these effects being rescued by telomerase or inactivation of p53 [28].

One possible explanation of these apparently conflictual results could be related to lamin A/C expression. Indeed, cells with low levels of lamin A/C are more sensitive to lamin B1 overexpression, displaying impaired proliferation, increased DNA damage, and senescence [29]. It is also conceivable that a range of lamin B1 amount is required to keep cells in a healthy condition, while altered *LMNB1* expression leads to senescence. Of note, it has been also shown that different senescence-inducing conditions as oncogene expression or oxidative stress elicit different effects on lamin B1 levels [30].

### Cell cycle regulation

B-type lamins play important roles in cell cycle regulation and cellular proliferation. In fact, B-type lamins are required to maintain chromatin condensation in interphase nuclei, and loss of lamin B1 is related to prolonged S phase [31]. Moreover, it has been demonstrated in cancer cells that a decrease of lamin B1 levels leads to a reduction of cell proliferation and deficiencies in DNA synthesis, resulting in the accumulation of cells in early S phase, probably due to a stalling of replication forks [32]. Indeed, it seems that lamin B1 may play an important role in the replication initiation, such as assembly of replicative complex, and in fork progression, especially during the elongation phase [32].

### DNA damage

It has been reported that lack of lamin B1 in cancer cells causes chromosomal instability and persistent DNA damages [32]. These damages were revealed by the numerous spontaneous γH2AX and 53BP1 foci, suggesting an accumulation of double-strand-breaks (DSBs). Depletion of lamin B1 induces variations of several repair proteins, including 53BP1, BRCA1, RAD50, and DNA-PKcs, NBS1, and RAD51 [32].

Moreover, lamin B1 seems to control the homologous recombination (HR) repair systems via its interaction with RAD51. It has been hypothesised that this interaction stabilizes RAD51 inhibiting its proteasomal degradation [33].

### Tissue development

Lamins B are ubiquitously expressed with high expression in different tissues as bone marrow, blood, gastrointestinal tract, and brain. As anticipated above, lamins B play a fundamental role in the normal development of different tissues, especially of the CNS [13, 23]. It follows that mutations in *LMNB* genes or alterations in lamin B levels have deleterious consequences and are linked to several CNS diseases.

First studies performed in murine models showed that lamin B1 plays a key role in embryonic development and organogenesis of brain [13, 34]. *LMNB1* knockout mice show perinatal lethality and are characterized by lower brain dimension, abnormal layering and apoptosis of cortical neurons [34].

In addition, lamin B1 is essential for dendrite differentiation in primary mouse cortical neurons [35], while lamin B2 localizes in axons and prevents axonal degeneration by maintaining mitochondrial function, axonal integrity and nuclear migration [36].

Loss of lamin B1 causes hypothalamic abnormalities, results in a decrease of cerebellar dimensions due to irregular neural migration [37], and may be related to nuclear membrane ruptures in migrating neurons, accompanied by DNA damage and cell death [38].

It has been recently reported that lamin B1 is downregulated with age in mouse hippocampal neural stem cells [39]. Importantly, restoring lamin B1 levels is sufficient to rescue proliferation deficits in aged mouse hippocampus, improving stem cell proliferation and CNS neurogenesis [39].

All the results support the thesis that a fine control of lamin B1 levels is critical for brain development and maintenance and a balanced expression of lamin B1 in neurons must be guarantee. Indeed, the overexpression of lamin B1 in the mouse CNS is related to abnormal neuronal activity, microglial reaction, astrogliosis and myelin abnormalities, including reduced synthesis of myelin in oligodendrocytes [40, 41]. Nevertheless, the specific mechanisms by which lamin B1 controls brain differentiation are still to be clarified.

## Lamin B-related laminopathies

Mutations in lamins or lamin-related genes lead to several human diseases called laminopathies that include muscle, metabolic, neuronal and ageing-related diseases. Even though most of the mutations are in the *LMNA* gene, mutations in *LMNB1* and *LMNB2* genes have been reported. The lower number of *LMNB*-related diseases compared to lamin A-related disorders is probably due to the fact that B-type lamins are required in embryonic and fetal development and gene mutations are often lethal. Moreover, lamin B expression levels are critical for protein functionality and weak changes in lamin B expression levels can lead to different disease, as reported below (Table 1).

**Table 1:** Lamin B-related laminopathies

Disease	Clinical phenotype	Gene	Lamin B misexpression	Biological phenotype	Refs.
Adult-onset Autosomal Dominant Leukodystrophy	Cerebral dysfunction, muscle weakness, spasticity autonomic dysfunction and cognitive impairment	*LMNB1*	Overexpression	Demyelination of the CNS	[83]
Lipodystrophy	Defects in the formation, distribution, and maintenance of adipose tissue	*LMNB2*	Mutation	Presence of autoimmune-mediated loss of adipose tissue	[54]
Epilepsy and Ataxia	Severe action myoclonus, progressive neurological decline, and ataxia	*LMNB2*	Mutation	Impaired neuronal migration	[58]
Ataxia Telangiectasia	Voluntary movements’ lack coordination, permanent widening of blood vessels	*LMNB1*	Overexpression	Demyelination of the CNS	[30]
Microcephaly	Brain developmental defect caused by significant smaller occipital–frontal head circumference	*LMNB1* *LMNB2*	Mutations	Weakness of nuclear lamina, impairied nucleokinesis and decreased neuronal survival	[60, 61]
Neural Tube Closure Tefects	Spina bifida and anencephaly	*LMNB1*	Mutation	Nuclear dysmorphology, altered cell cycle progression and premature senescence	[62]
Alzheimer’s Disease	Neurodegeneration	*LMNB1*	Decrease of expression	Pathological tau decreases lamin B causing relaxation of constitutive heterochromatin that causes neuronal death	[63]
Greenberg Dysplasia	Skeletal dysplasia, fetal hydrops, short limbs, and abnormal chondro-osseous calcification	LBR	Mutations	Defects in sterol metabolism caused by LBR mutations	[80]
Pelge–Huet Anomaly	Epilepsy, developmental delay, and skeletal abnormalities	LBR	Mutations	Altered shape of nuclei in granulocytes neutrophils and eosinophils	[82]

### Adult-onset autosomal dominant leukodystrophy (ADLD)

Adult-onset Autosomal Dominant Leukodystrophy (ADLD) is characterized by accumulation of lamin B1 caused by tandem duplications involving the *LMNB1* gene or deletions upstream of the *LMNB1* gene [42, 43]. ADLD is an extremely rare disease characterized by late onset neurological features, such as progressive myelin loss in the CNS and fatal outcome [42]. In the majority of ADLD cases, the first clinical manifestations are related to autonomic dysfunction, followed by ataxia and cognitive impairment that reflects pyramidal and cerebellar involvement.

Several studies demonstrated that *LMNB1* gene duplication is related to defects of brain development and to a severe demyelinating phenotype [42]. Of note, lamin B1 has to be finely tuned during brain development, with a peak at birth and followed by a successive gradual decrease [44].

Lamin B1 overexpression affects inner nuclear membrane proteins, chromatin organization, and nuclear pore transport, causing defects in oligodendrocytes differentiation [44, 45]. *LMNB1* duplication is also related to inhibition of myelin-specific genes and to the activation of glial fibrillary acidic protein (GFAP) transcription [44].

An increase of lamin B1 expression is also related to an untimely block of oligodendrocytes’ differentiation characterized by an alteration of the myelin proteolipid protein (PLP), the major myelin membrane lipoprotein [44]. Importantly, different types of mutations in *PLP1* gene leads to demyelination in a group of disorders known as hypomyelinating leukodystrophies (Pelizaeus-Merzbacher diseases) [46], while PLP overexpression causes oligodendrocytes death [47].

Different mouse models have been developed to better understand the biological mechanisms that link lamin B1 overexpression and demyelination in ADLD patients. In 2013, Heng et al. generated a BAC transgenic mouse model carrying additional copies of murine wild-type lamin B1 (*Lmnb1*^BAC^) that recapitulates several features of ADLD [40]. The same group created transgenic mice overexpressing *Lmnb1* in different CNS cell lineages, demonstrating that lamin B1 overexpression in oligodendrocytes is sufficient for the onset of histopathological, molecular, and behavioural deficits characteristic of ADLD (not in neurons or astrocytes). Moreover, they reported that lamin B1 overexpression induces motor deficits, aberrant myelin formation, axonal degeneration, and demyelination with a marked decrease of PLP1, due to reduced occupancy of the transcription factor Yin Yang 1 (Yy1) at the promoter [40].

A following independent study generated an ADLD derived oligodendrocyte-specific transgenic mouse overexpressing lamin B1 [41]. This interesting study demonstrated a molecular link between lamin B1 and lipid synthesis in oligodendrocytes and a reduction of myelin-enriched lipids associated to myelin destruction at spinal cord level [41].

Significant insights into the pathophysiology of ADLD have been provided by Bartoletti-Stella and al. that investigated how lamin B1 duplication may impact the whole-genome expression profile, in tissues derived from ADLD patients [48]. Authors reported that duplication of *LMNB1* affects transcription and alternative splicing of several genes associated with the immune system, neuronal and skeletal development. Among these genes, raver2, a RNA-binding protein (RBP) that modulates the splicing repressor polypyrimidine tract binding protein (PTB), is significantly increased [48].

Although the demyelination is one of the most significant aspects of ADLD, the molecular and functional mechanisms underlying this pathology have not been fully elucidated. About this, Bartoletti-Stella et al. suggest that ADLD could be considered as a “spliceopathy” caused by increased levels of lamin B1 and raver2 and that the characteristic demyelination could be the result of increased expression, during adulthood, of the embryonic isoform of the PLP1 protein, which has a crucial role in myelin maintenance. Of note, the pivotal role of raver2 in brain development was previously demonstrated in the brains of Lmnb1Δ/Δ embryos, where a dysregulation of raver2 may cause abnormal development, with reduced brain size and impaired corticogenesis [13, 34, 49].

Recent studies indicated that also astrocytes may be pivotal in the development of ADLD. In particular, our research group demonstrated that several nuclear alterations are found in astrocytes, but not in oligodendrocytes, that overexpress lamin B1 [50]. These astrocytes are characterized by reduction in leukaemia inhibitory factor (LIF) secretion which, in turn, leads to down-regulation of the pro-survival Janus kinase-signal transducer and activator of transcription protein 3 (Jak/Stat3) and phosphatidylinositol-3 phosphate kinase (PI3K)/Akt signalling pathways. Increased production of ROS is found in primary dermal fibroblasts from ADLD patients, demonstrating that they may play an additional role in the pathogenesis of ADLD [50]. This effect is consistent with previous studies showing altered nuclear dynamics of the transcription factor Oct-1 in ADLD dermal fibroblast [51]. Oct-1, a main player in stress response, is sequestered at the nuclear envelope by overexpressed lamin B1, which causes altered stress response [51]. The involvement of muscle tissue in ADLD due to impairment of myosin heavy chain 7 expression downstream of Oct-1 dysregulation has been also suggested [51].

A link between lamin B1 overexpression and signalling pathway alterations has been recently supported by our last article, reporting that lamin B increase leads to inactivation of glycogen synthase kinase (GSK)3β, but not the upregulation of β-catenin targets and to a reduction of astrocyte survival in vitro [52]. Interestingly, astrocytes overexpressing lamin B1 show increased immunoreactivity for both GFAP and vimentin together with NF-κB phosphorylation and c-Fos increase, suggesting astrocytes reactivity and substantial cellular activation [52].

Finally, a recent study has achieved to abrogate the ADLD-specific phenotypes with the use of siRNA treatment (non-duplicated allele by ASP-siRNA used may reduce the amount of *LMNB1* without excessively downregulating the gene) in ADLD fibroblasts, in murine oligodendrocytes overexpressing human *LMNB1*, and neurons directly reprogrammed from patients' fibroblasts, providing an effective therapeutic strategy for ADLD treatment [53].

### Lipodystrophies

Lipodystrophies are a heterogeneous group of genetic or acquired diseases characterized by defects affecting formation, distribution, and maintenance of adipose tissue. Lipodystrophic patients show a loss of fat in specific district or in the whole body. Mutations in *LMNB2* may cause susceptibility to acquired partial lipodystrophy. Indeed, enrichment of heterozygous *LMNB2* variants in acquired partial lipodystrophy patients has been described [54, 55].

### Lamin B-related neuronal disorders

Several lamin B-related laminopathies are frequently confined to CNS. Unlike lamin A, which is physiologically expressed at low levels in the brain, *LMNB1* gene is highly expressed and small fluctuations in the expression levels seem to have remarkable molecular and functional consequences for the CNS cells [29, 56]. Of note, both lamin B1 and B2 are required during brain development as they regulate nuclear migration during neurogenesis of the CNS.

In murine and human models, indeed, mutations or alterations of lamin B expression are related to different neuronal diseases (as reported below). Mice carrying homozygous deletions of *Lmnb1* and *Lmnb2* genes die at birth and display a neuronal phenotype resembling lissencephaly [13, 37, 57]. Moreover, *Lmnb2*-null mice showed abnormal development of the cerebral cortex and cerebellum [34, 57].

- Epilepsy and ataxia
  Variants of *LMNB2* have been described in a family with progressive myoclonic epilepsy and ataxia [58]. Homozygous missense mutation in *LMNB2* has been found in patients with autosomal recessive progressive myoclonus epilepsy with early ataxia syndrome, probably caused by a mistaken neuronal migration.
- Ataxia telangiectasia
  Similar to ADLD, elevated levels of lamin B1 have also been reported in patients with ataxia telangiectasia (AT), an autosomal-recessive disorder characterized by neurological defects, including demyelination of the CNS [30]. AT is caused by mutations of ataxia telangiectasia mutated (ATM) that controls early steps during DNA damage response signalling and could contribute to cellular senescence [28, 59]. The complex relationship between elevated lamin B1 levels and impaired DNA damage response has been previously reported.
- Microcephaly
  Microcephaly has been related to dominant lamin B1 and B2 variants [60, 61]. In human neurons, where lamin B1 is more abundant, impaired nuclear envelope integrity can result in a spectrum of negative consequences that ultimately lead to microcephaly in affected individuals. These variants appear to interfere with the normal assembly of the nuclear envelope, altering the conformation of lamin B1, weakening the nuclear lamina, impairing nucleokinesis and leading to decreased neuronal survival [38].
  Furthermore, the alteration of nuclear envelope integrity, as observed for these different variants, influences the survival and migration of neural precursors, leading to a marked reduction of brain size.
- Neural tube closure defects
  A *LMNB1* polymorphic variant has been related to neural tube closure defects, including spina bifida and anencephaly [62]. This variant displays a decreased stability of the nuclear lamina, resulting in increased nuclear dysmorphology, altered cell cycle progression and premature cellular senescence.
- Alzheimer’s disease
  A marked reduction of lamins B expression has been reported in cortical neurons of Alzheimer’s disease (AD) patients [63]. In particular, in neurons of AD patients, pathological tau leads to the stabilization of actin filaments, disrupting the Linkers of the nucleoskeleton to the cytoskeleton (LINC) complex and decreasing lamin B. The consequences are the relaxation of constitutive heterochromatin and the activation of cell cycle in post-mitotic neurons, causing the death of the neurons [64]. The involvement of lamin B in AD onset suggests that this widespread neurodegenerative disorder may be considered as an aged-related laminopathy [64, 65].

## Cancer

The aberrant localization or expression of lamins B has been related to cancer development, aggressiveness and metastasis, with lamin B being misregulated in a wide variety of cancers, probably because of the deformation of nuclear morphology caused by lamins B alterations [66] (Table 2). Indeed, cancer cells are subjected to changes of nuclear shape that favour the capability of cells to migrate and hence to promote metastasis. Given that lamins provide structural and mechanical strength as well as stiffness to the nucleus, their role in tumour development is now under growing and attentive examination, even if it is still under investigations whether lamins B misexpression modulate the ability of cancer cells to metastasize by altering the nucleus mechanical properties or rather by acting directly on cell proliferation, signalling, and differentiation.

**Table 2:** Lamin B involvement in human cancer

Cancer type	Gene		Lamin B misexpression	Refs.
Pancreatic	LMNB1		Overexpression	[67]
Liver	LMNB1		Overexpression	[68]
		LMNB2	Overexpression	[75]
Lung	LMNB1		Decrease	[72]
		LMNB2	Overexpression	[76]
Breast	LMNB1		Decrease	[71]
		LMNB2	Overexpression	[77]
Colon	LMNB1		Decrease	[70]
		LMNB2	Overexpression	[88]

It has been demonstrated that lamin B1 and lamin B2 are up- and down-regulated in several cancer cells and, accordingly, play different roles in cancer onset and development (Table 2). However, a particular interest should be addressed to lamin B1 expression, because there are some contradictory results when we take into account different types of tumour. While some study reported that increased lamin B1 expression is related to a more aggressive and invasive phenotype (in pancreatic and liver cancer [67, 68]) and importantly, that overexpression of lamin B1 is accompanied by a bad prognosis (in renal and gastric cancers [69, 70]), a decrease of lamin B expression has been also associated with greater aggressiveness and bad prognosis (in lung and breast cancer [71, 72]). These contradicting results suggest that the numerous functions that lamins B carry out in the cell may have different effects in cancers that arise from different cell types. Moreover, the interactions of lamin B with other binding proteins and signalling downstream components have been found in several tumour cells, contributing to the cancer development.

A possible involvement of lamin B in cancer onset could be related to the relationship between lamin B and oxidative stress, which is in turn connected with aggressiveness and metastasis [73]. In fact, it has been demonstrated that lamin B1 is overexpressed under oxidative stress conditions [30]. Another explanation of the role of lamin B in carcinogenesis could be that the overexpression of lamin B1 can hasten cell migration through the inhibition of the formation of a perinuclear actin rim that typically limits cell migration rate, as recently demonstrated [74].

However, further investigations to unravel the specific role of lamins B alterations in the different forms of cancer are needed, hence providing new insights in better understanding the cancerogenic processes regulated by variations of these type of lamins.

- Pancreatic cancer
  Aberrant expression of lamin B1 has been identified in pancreatic cancer cells and related to a more aggressive phenotype with worse prognosis [67]. For this reason, it has been suggested that the increase of lamin B1 could be considered as a novel biomarker for pancreatic cancer and as a therapeutic target [67]. On the contrary, the decrease of lamin B1 reduces the proliferation, invasion, and tumorigenicity of pancreatic cancer cells [67]. This is particularly interesting as there are several inhibitors of pre-lamin B1 processing at the level of farnesylation or methylation, which could be explored as modulators of lamin B1 levels.
- Liver cancer
  It has reported that lamin B1 expression increases in hepatocellular carcinoma (HCC) cells [68]. Moreover, it has been recently reported that also lamin B2 is involved in HCC cancer development promoting cell proliferation, migration, and invasion in HCC cell lines and in primary HCC cells [75].
- Lung cancer
  Recently, a link between lung cancer onset and lamins B has been demonstrated. Deletion of one copy of *LMNB1* gene in mice and in human is related [72]. Lower levels of lamin B1 expression, indeed, in patients affected by lung cancer favour metastasis through epigenetic mechanism [72]. In particular, lamin B1 acts as onco-suppressor recruiting the polycomb repressive complex 2 (PRC2) and repressing genes involved in cell migration, including RET and p38 MAPK signalling axis components. From this perspective, lamin B1 plays a key role in lung cancer progression, providing a molecular link between altered nuclear morphology, aberrant epigenetic patterning, and malignant phenotype.
  Furthermore, Zhang et al. reported that the overexpression of lamin B2 promotes migration of non-small cell lung cancer by increasing H3K9me2 level, thus eliciting E-cadherin gene silencing [76].
- Breast cancer
  In breast cancer, different studies have reported that both a decreased expression of lamin B1 [71] and an increase of lamin B2 [77] are related to bad outcome, confirming the importance to strictly maintain proper B-type lamin levels.
- Neoplasms of the gastrointestinal tract
  Decrease of lamin B1 expression is linked to different type of gastrointestinal tumours, including colon carcinomas, adenomas and gastric cancers [70, 78].

## Lamin B receptor diseases

Another spectrum of pathologies connected to lamin B alterations involve mutations in the lamin B receptor (LBR). The LBR is an inner nuclear membrane protein that binds lamin B proteins (both lamin B1 and B2). Two different functions have been attributed to LBR. The nucleoplasmatic domain interacts with chromatin and has a structural function, whereas its transmembrane domains exhibit sterol reductase activity, a crucial function of the cholesterol synthesis metabolic pathway (Further details on LBR in the review of Nikolakaki et al. [79]).

Mutations of LBR have been related to two rare disease: Greenberg dysplasia and Pelger–Huet anomaly; the former is a lethal disease characterized by skeletal dysplasia, fetal hydrops, short limbs and abnormal chondro-osseous calcification [80], the latter is an autosomal dominant pathology, characterized by altered shape of nuclei in neutrophil and eosinophil granulocytes [81, 82], which is asymptomatic in the heterozygous state and causes variable degrees of epilepsy, developmental delay and skeletal abnormalities in homozygotes [82].

## Conclusions and perspectives

Growing amount of evidence shows that B-type lamins play an important role in laminopathies and cancer. Besides the clear involvement of B-type lamins in CNS diseases and the fact that *LMNA*-linked disorders do not affect brain functionality, several aspects of lamin B-related pathophysiology highlight the specificity of B-type lamins in cellular and organism homeostasis with respect to A-type lamins. The pathogenesis of ADLD, the main lamin B-linked disease, has been so far linked to increased ROS levels and altered lipid metabolism leading to demyelination [83]. The association of lamin B2 with APL further supports a role for B-type lamins in lipid and adipose tissue metabolism. Yet, the molecular mechanisms can be recapitulated into a few pathways involving chromatin anchorage at specific sites (lamina-associated domains, LADs), a group of repressed sequences that could be reactivated in the presence of altered lamin B levels. Elevated ROS levels caused by increased lamin B1-Oct-1 interaction and altered nuclear dynamics has been also suggested as a pathogenetic pathway for ADLD and ADLD pathogenesis has been further linked to the onset of aberrant splicing events elicited by elevated lamin B1 levels [27, 51].

A clear input for further research that could also open therapeutic perspectives is the well corroborated evidence that lamin B amount is a determinant of protein functionality. In fact, while increased lamin B1 levels are linked to ADLD, reduced lamin B1 amount is associated with ageing processes. On the other hand, depending on the trigger, cellular senescence can be also linked to increased lamin B1 amount [30], a condition recently linked to sequestering of the DNA damage repair factor 53BP1 and altered DNA damage response [84].

Even in cancer cells, levels of B-type lamins are altered and a relationship between cancer aggressiveness and lamin amount can be established in a tumour-specific way. These observations imply that drugs affecting lamin B1 post-translational processing, including isoprenylcysteinecarboxyl methyltransferase (ICMT) or RCE1 inhibitors could be explored for their effect on lamin B stability in view of therapeutic approaches [85–87].

## Data Availability

Not applicable.
